# Proof-of-Principle Study of Inflammasome Signaling Proteins as Diagnostic Biomarkers of the Inflammatory Response in Parkinson’s Disease

**DOI:** 10.3390/ph16060883

**Published:** 2023-06-15

**Authors:** Erika d. l. R. M. Cabrera Ranaldi, Karen Nuytemans, Anisley Martinez, Corneliu C. Luca, Robert W. Keane, Juan Pablo de Rivero Vaccari

**Affiliations:** 1 Department of Neurological Surgery and The Miami Project to Cure Paralysis, University of Miami Miller School of Medicine, Miami, FL 33136, USA; 2 The Dr. John T. Macdonald Foundation, Department of Human Genetics, University of Miami Miller School of Medicine, Miami, FL 33136, USA; 3 John P. Hussman Institute for Human Genomics, University of Miami Miller School of Medicine, Miami, FL 33136, USA; 4 Department of Neurology, University of Miami Miller School of Medicine, Miami, FL 33136, USA; 5 Department of Physiology and Biophysics, University of Miami Miller School of Medicine, Miami, FL 33136, USA

**Keywords:** inflammasome, biomarkers, caspase-1, ASC, Parkinson’s disease

## Abstract

Parkinson’s disease (PD) is a neurodegenerative disorder marked by the death of dopaminergic neurons in the midbrain, the accumulation of α-synuclein aggregates, and motor deficits. A major contributor to dopaminergic neuronal loss is neuroinflammation. The inflammasome is a multiprotein complex that perpetuates neuroinflammation in neurodegenerative disorders including PD. Increases in inflammasome proteins are associated with worsened pathology. Thus, the inhibition of inflammatory mediators has the potential to aid in PD treatment. Here, we investigated inflammasome signaling proteins as potential biomarkers of the inflammatory response in PD. Plasma from PD subjects and healthy age-matched controls were evaluated for levels of the inflammasome protein apoptosis-associated speck-like protein containing a caspase recruitment domain (ASC), caspase-1, and interleukin (IL)-18. This was carried out using Simple Plex technology to identify changes in inflammasome proteins in the blood of PD subjects. The area under the curve (AUC) was obtained through calculation of the receiver operating characteristics (ROC) to obtain information on biomarker reliability and traits. Additionally, we completed a stepwise regression selected from the lowest Akaike information criterion (AIC) to assess how the inflammasome proteins caspase-1 and ASC contribute to IL-18 levels in people with PD. PD subjects demonstrated elevated caspase-1, ASC, and IL-18 levels when compared to controls; each of these proteins were found to be promising biomarkers of inflammation in PD. Furthermore, inflammasome proteins were determined to significantly contribute to and predict IL-18 levels in subjects with PD. Thus, we demonstrated that inflammasome proteins serve as reliable biomarkers of inflammation in PD and that inflammasome proteins provide significant contributions to IL-18 levels in PD.

## 1. Introduction

Parkinson’s disease (PD) is a neurodegenerative disorder where dopaminergic neurons are lost and α-synuclein oligomers accumulate, manifesting clinically as parkinsonism. Individuals with PD experience widespread symptoms, including resting tremor, rigidity, bradykinesia, and in the later stages, cognitive impairment such as memory loss and dementia [1]. Lewy bodies and Lewy neurites are pathological inclusions which congregate within the neuronal cytoplasm in PD [2]. Oligomerized aggregates of α-synuclein are major contributors to PD pathology, spreading from cell to cell and seeding further α-synuclein accumulation and Lewy body formation in other neurons [3,4]. Additionally, α-synuclein pathology can contribute to neuronal loss in the substantia nigra pars compacta (SNpc) and striatum [3], resulting in motor deficits. However, despite PD being the second most prevalent neurodegenerative disorder, clinical diagnostic models fall short and there are no currently accepted biomarkers for the condition. Furthermore, during the earlier stages of the disease, it is difficult to distinguish between PD and alternative parkinsonian conditions, such as multiple system atrophy, making accurate diagnosis even more challenging [1]. Thus, there is a need for biomarkers that detect the disease early on, distinguish PD from other conditions, and provide guidance on disease progression to tailor improved treatments.

Interestingly, an important part of PD pathology is immune activation and neuroinflammation in the central nervous system (CNS) [5]. Microglia are critical immune modulators which are activated by danger-associated molecular patterns (DAMPs) and pathogen-associated molecular patterns (PAMPs) binding to pattern-recognition receptors (PRR) on the microglia’s surface and cytoplasm [6,7,8]. Stimulation of PRRs, such as toll-like receptors (TLRs), prime the activation of the inflammasome, a critical immune-modulating complex of receptors and sensors [9,10]. Activation of the inflammasome leads to the recruitment of apoptosis-associated speck-like protein containing a caspase recruitment domain (ASC), an adaptor protein with the ability to oligomerize into filamentous aggregates known as ASC specks. ASC specks recruit and activate pro-caspase-1 [11]. The activated caspase-1 hydrolyzes the pro-inflammatory cytokines interleukin (IL)-1β and IL-18 [10,12,13,14]. Moreover, caspase-1 also mediates gasdermin-D cleavage, leading to pyroptotic cell death through the formation of membrane pores, which can further release inflammatory molecules [13,15].

In PD animal models, specifically those models with the administration of lipopolysaccharide (LPS), 6-hydroxydopamine (6-OHDA), and 1-methyl-4-phenyl-1,2,3,6-tetrahydropyridine (MPTP), elevated NOD-like receptor protein 3 (NLRP3) inflammasome activation results in worsened motor dysfunction and dopaminergic neuronal loss in the SNpc [16,17,18]. Specifically, caspase-1 reduction via knockdown or inhibition rescues neuronal death [18,19]. Furthermore, post-mortem tissue samples from people with PD exhibit elevated caspase-1 and ASC levels in the substantia nigra (SN) [16], and oligomeric α-synuclein can prime and activate the NLRP3 inflammasome [20,21], leading to the perpetuation of neuroinflammation via the release of pro-inflammatory cytokines such as IL-1β and tumor necrosis factor (TNF)-α [22,23]. In contrast, inflammasome inhibition facilitates clearance of α-synuclein oligomers and reduces related pathology [16,17,24]. In addition, several genetic mutations have been linked to an increased incidence of PD. For instance, *SNCA* and *LRRK2* [25] mutations are associated with autosomal dominant presentations of PD, the former being affiliated with α-synuclein aggregation in PD, while the latter encodes for the LRRK2 protein, involved in immune regulation [25,26]. In addition, autosomal recessive mutations such as *Parkin*, *PINK1*, and *DJ-1* are also affiliated with PD development [25]. Such genetic changes can modulate innate immune responses and gliosis [27,28,29]. For example, Parkin deficiency results in enhanced NLRP3 inflammasome activation and pro-inflammatory cytokine release in primary mouse microglia [28].

In PD subjects, approximately 30% of SN dopaminergic neurons are lost by the time motor symptoms are detected [30]. With an estimated incidence rate of nearly 90,000 cases of PD per year in individuals aged 65 years and older in North America [31], the importance of developing early detection mechanisms that can serve to diagnose patients before irreversible damage occurs is critical. Previous studies have established the relationship between PD and inflammasome activity; thus, characterization of the potential for inflammatory molecules to be used as biomarkers for PD detection is critical.

In the present study, we investigated levels of inflammasome-associated proteins and the downstream pro-inflammatory cytokine IL-18 in PD subjects compared to age-matched unaffected controls. The receiver operating characteristic (ROC) curve was determined for each potential biomarker. Furthermore, sensitivity and specificity were characterized and the area under the curve (AUC) was used to identify which inflammatory analyte could have the greatest reliability for clinical use. Additionally, a stepwise regression was carried out to predict the contribution of inflammasome signaling proteins to levels of IL-18 in PD subjects.

## 2. Results

### 2.1. PD Patients Have Increased Plasma Levels of Inflammasome Proteins and Inflammatory Cytokines

**Figure 1:**
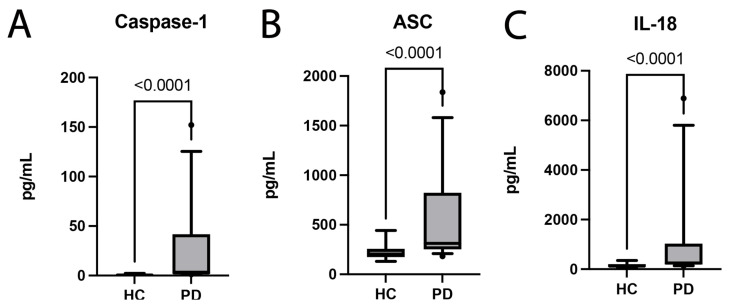
**Inflammasome Components and Pro-Inflammatory Cytokines are Increased in PD subjects.** The Simple Plex assay was utilized to quantify inflammasome proteins in the plasma of patients with PD and healthy age-matched controls. Inflammasome proteins, (**A**) caspase-1 and (**B**) ASC, were significantly elevated in patients with PD versus age-matched healthy controls. (**C**) The inflammatory cytokine IL-18 was also significantly increased in patients with PD when compared to healthy controls (HC). (**A**) caspase-1: N: control: 13, PD: 32; (**B**) ASC: N: control: 16, PD: 32; (**C**) IL-18: N: control: 16, PD: 32.

The inflammasome has been previously implicated in the pathology of PD [18]. First, we evaluated the levels of inflammasome proteins in plasma samples of patients with PD and healthy age-matched controls to determine the levels of inflammasome proteins in these subjects. Accordingly, we determined levels of the inflammatory proteins caspase-1 (Figure 1A), ASC (Figure 1B), and IL-18 (Figure 1C) in plasma samples of patients with PD and healthy age-matched controls. PD subjects had significantly higher levels of all three inflammasome proteins in their plasma when compared to healthy controls. Overall, the results provide evidence of an increased pro-inflammatory response in PD that is, in part, contributed to by the inflammasome in humans.

### 2.2. Inflammasome Proteins Are Reliable Biomarkers of PD

**Figure 2:**
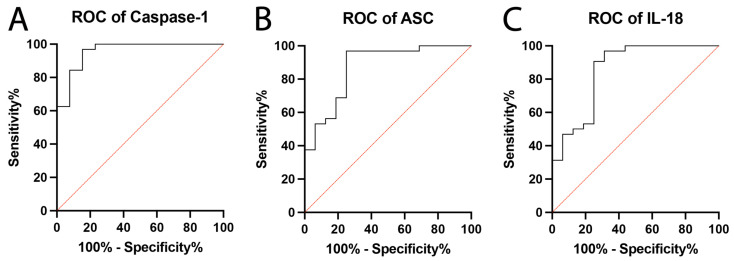
**Calculated ROC for Inflammatory Biomarkers of PD:** The ROC and AUC were calculated for caspase-1, ASC, and IL-18, as they were previously identified to be significantly elevated in patients with PD when compared to healthy controls. (**A**) Caspase-1: N: control: 13, PD: 32; (**B**) ASC: N: control: 16, PD: 32; (**C**) IL-18: N: control: 16, PD: 32.

It has been previously shown that the NLRP3 protein of the inflammasome is elevated in patients with PD [32]. Thus, we aimed to determine the inflammatory biomarker potential of inflammasome signaling proteins by measuring the levels of caspase-1, ASC, and of the pro-inflammatory cytokine IL-18. We evaluated their reliability as inflammatory biomarkers of PD by plotting the ROC curve for each protein. Caspase-1 (Figure 2A) had the highest AUC value of the inflammasome proteins examined at 0.96 (Table 1) with a specificity of 85% and a sensitivity of 96.88% (Table 2). ASC (Figure 2B) also had a high AUC value (0.87) with a specificity of 75% and a sensitivity of 96.88%. The inflammatory cytokine IL-18 (Figure 2C) demonstrated a high AUC value (0.85) with a specificity of 75% and a sensitivity of 96.88%. Overall, the results indicate that all three inflammatory proteins could act as reliable biomarkers of the inflammatory response in PD subjects.

### 2.3. Caspase-1 and ASC Contribute to the Protein Levels of IL-18

In order to determine whether the inflammasome proteins caspase-1 and ASC contributed to IL-18 levels, we conducted a multivariate linear regression that was fitted using a stepwise approach. The best model was determined by identifying the lowest AIC. Following the stepwise method, the standard error, estimate (coefficients), and *p*-values for each of the inflammatory proteins and the intercept (slope) were calculated (Table 3). Additionally, based upon all identified inflammasome protein biomarkers, the BIC (527.84), confidence intervals, DW autocorrelation, RMSE (781.52), and mean of residuals (4.88 × 10^−15^) (Figure S1) were determined for the best fit model:

IL-18 = 907.19 + 60.67 (caspase-1) − 2.79 (ASC)

Model: IL-18 = 907.19 + 60.67 (caspase-1) − 2.79 (ASC)

Together, as characterized by the best fit model, we established that caspase-1 and ASC contributed to IL-18 levels, with an adjusted R^2^ of 0.78 and *p*-value of 1.366 × 10^−10^. This indicates that, in this cohort of patients, caspase-1 and ASC contribute to 78% of the protein levels of IL-18 in the plasma.

## 3. Discussion

In this study, we identified significant increases in caspase-1, ASC, and IL-18 protein levels in patients with PD, compared with controls. Each protein demonstrated robust specificity, sensitivity, and AUC values indicating that they are potential reliable biomarkers of the inflammatory response in PD. Additionally, using a linear regression analysis, we observed that inflammasome signaling proteins significantly contribute to and predict IL-18 levels in patients with PD.

Despite PD being the second most common neurodegenerative disorder, clinical diagnostic models fall short and there are no currently accepted biomarkers for this disorder. Biomarkers currently under investigation include non-invasive methods such as brain imaging or cerebrospinal fluid (CSF) and blood biomarkers. Brain imaging modalities are currently being investigated for PD detection. Reductions in dopaminergic neuronal integrity in the SN and striatum can be examined by monitoring ^18^F-dopamine [33] and ^18^F-FE-PE2I [34] activity with positron emission tomography and can discriminate between PD and healthy controls. Additionally, various studies have investigated the potential of fluid biomarkers for PD monitoring, with great focus on α-synuclein levels. Some studies have indicated that total α-synuclein levels are reduced in the CSF of patients with PD compared to the values of healthy controls and non-parkinsonian conditions such as Alzheimer’s disease [35,36]. However, one meta-analysis of 34 studies concluded that CSF concentrations of α-synuclein species in PD do not significantly differ when compared to those in other parkinsonian conditions, with poor specificity for both total and oligomeric forms of α-synuclein [37]. Alternatively, peripheral immune cells are another investigated target; for example, peripheral blood mononuclear cells isolated from patients with PD release greater amounts of pro-inflammatory cytokines, which are associated with greater disease dysfunction [38].

The serum of patients with PD has been found to contain increased pro-inflammatory cytokines such as tumor necrosis factor (TNF) and IL-1β [39,40]. Additionally, IL-1β administration elevates α-synuclein protein expression in rat primary neuronal cultures and increases α-synuclein mRNA expression in rat cortices [41]. Further, chronic expression of IL-1β in the rat SNpc results in the death of dopaminergic neurons, immune cell infiltration, glial activation, and motor deficits [42], linking the elevation of pro-inflammatory cytokines to PD-like pathology in vivo. Oligomerized α-synuclein can act as a DAMP and activate microglia by binding to TLRs on their surface, leading to morphological changes, and the release of IL-1β and TNF [23,24].

Microglial activation driven by α-synuclein also exacerbates dopaminergic neuronal toxicity in primary mesencephalic cultures [43]. DAMPs such as α-synuclein can also activate the inflammasome; the addition of α-synuclein to murine microglial cells can activate NLRP3 protein expression and lead to ASC speck formation [16], in addition to IL-1β release through caspase-1 signaling [40]. Interestingly, NLRP3 inflammasome activation modulates dopaminergic neuronal cell death via pyroptotic mechanisms [44], and NLRP3 inflammasome inhibition can rescue dopaminergic neuronal loss, improve motor dysfunction, and alleviate neuroinflammation in rodent models of PD [16,18,45,46,47]. In humans, serum levels of NLRP3 are elevated in patients with PD and are associated with greater total and phosphorylated α-synuclein levels [48], thus providing further evidence of the interplay between the inflammasome and PD pathology. Moreover, post-mortem mesencephalic tissue of patients with PD demonstrates increased NLRP3 and ASC immunoreactivity, as well as NLRP3 co-localization with dopaminergic neurons [32] and Lewy body neurites [49]. Importantly, reductions in inflammasome activity via NLRP3 deficiency or inhibition reduce microglial activation and protect against dopaminergic neuronal loss in the SNpc in the MPTP [40] and rotenone mouse models of PD [50]. In addition, caspase-1 inhibition also reduces inflammasome mRNA expression [18] and reverses motor deficits and neuronal loss in PD mouse models [16,18]. Furthermore, an *NLRP3* genetic polymorphism that affects NLRP3 stability and increases its degradation has been associated with a significantly lower risk of developing PD [49]. Hence, since there is considerable interaction between the inflammasome and PD pathology, it appears that inflammasome signaling proteins could function as promising biomarkers of the inflammatory response present in patients with PD.

However, despite studies establishing heightened inflammasome activation in patients with PD, few studies have examined the biomarker potential of such molecules for diagnostic and prognostic purposes. Importantly, it is necessary to determine the ROC curve for each potential biomarker in order to obtain the AUC value and properly establish biomarker characteristics; an AUC value of 1 characterizes a biomarker with perfect capability for detection, and values above 0.8 are widely understood to be reliable [51]. In our study, microfluidics technology was used to identify inflammasome signaling proteins and pro-inflammatory cytokine biomarkers. ROC curves plotting biomarker sensitivity and specificity were obtained, and cut-off points that maximized sensitivity and specificity values were additionally selected. According to our statistical analyses, the inflammasome proteins caspase-1 and ASC were found to be reliable biomarkers with high AUC values of 0.96 and 0.87, respectively. For the pro-inflammatory cytokine, IL-18 also demonstrated a high AUC value of 0.85. The candidate biomarkers also presented high sensitivity. Namely, caspase-1 and ASC presented a 96.88% sensitivity, indicating a dependable capability for the detection of PD-related inflammation. IL-18 also presented a high sensitivity of 90.63%. As for specificity, caspase-1 had the highest specificity at 85%, followed by ASC and IL-18 at 75% each. Moreover, accuracy for caspase-1 and ASC was 93% and 90%, respectively, and 85% for the pro-inflammatory cytokine IL-18. Thus, the inflammasome signaling proteins and inflammasome-associated pro-inflammatory cytokine IL-18 have demonstrated great reliability in distinguishing the inflammatory response in patients with PD when compared to age-matched, unaffected controls. Consequently, they represent robust candidate biomarkers. Additionally, our study established the biomarker potential and characteristics of inflammatory proteins in blood plasma samples, which provide greater accessibility through a less invasive procedure that has a reduced contamination risk compared to other fluid biomarker sources such as CSF. However, future studies should aim to correlate inflammasome biomarkers in CSF with those in plasma in order to identify the nature of the relationship between the proteins found in CSF and those in the peripheral blood circulation. Here, we also investigated whether inflammasome proteins predict IL-18 levels in patients with PD. IL-18 is a mediator of local inflammation and induces interferon-γ production in natural killer cells and T-cells [52]. Caspase-1, ASC, and IL-18 levels are increased in patients with PD, suggesting that inflammasome proteins play a role in the inflammatory response. This finding is consistent with previous studies showing that inflammasome proteins are promising biomarkers of the inflammatory response associated with traumatic brain injury [53,54,55,56,57,58], stroke [59], Alzheimer’s disease [60], multiple sclerosis [61], macular degeneration [62], ocular surface damage [63], male pattern baldness [64], psoriasis [65], depression [66], renal diseases [67], and non-alcoholic steatohepatitis [68].

Interestingly, when comparing the cut-off points in this study for the inflammasome proteins caspase-1 (1.450 pg/mL), ASC (222.5 pg/mL), and IL-18 (159.5 pg/mL) with the cut-off points for the same proteins in patients with multiple sclerosis [61], we found that the inflammatory response contributed by the inflammasome was higher in patients with MS (caspase-1: 1.776 pg/mL, ASC: 537.5 pg/mL, IL-18: 238.2 pg/mL) [61] than in patients with PD.

The goal of this project was to identify cut-off points for inflammatory biomarkers of PD. Since the inflammasome contributes to the inflammatory response in PD, it is important to identify diagnostic, prognostic, and theragnostic markers of the disease. This study represents a first step towards identifying those biomarkers that can be used in the care of patients with PD. Hence, inflammasome proteins as biomarkers cannot differentiate PD from other neurodegenerative diseases such as Alzheimer’s disease or multiple sclerosis (MS). However, in this study, we identified that inflammasome proteins can be considered as biomarkers for the inflammatory response associated with PD, and in the future, this information could be used to monitor response to treatment. Thus, future studies should aim to measure inflammasome proteins as theragnostic biomarkers of PD. Moreover, while our study and previous research has demonstrated increased levels of inflammasome components in patients with PD compared to healthy controls, there is little information available regarding whether inflammasome biomarkers can distinguish between PD and other neurodegenerative diseases. However, mesencephalic tissues of patients with PD have greater immunoreactivity for inflammasome components compared to healthy controls, and patients with PD also differ in their ASC and NLRP3 expression when compared to individuals with non-PD nigral cell loss [32]. This demonstrates a gradient of expression that could separate PD from non-PD mesencephalic degeneration. In relation to NLRP3 expression, miR-223-3p—which downregulates NLRP3 activity—shows differential expression in the serum of patients with Alzheimer’s disease and mild cognitive impairment compared to those with PD [69]. Furthermore, pro-inflammatory cytokine levels, such as IL-1β in the CSF, can distinguish between PD and multiple system atrophy as well as progressive supranuclear palsy [70]. Therefore, inflammasome components show promise in discriminating between PD and other neurodegenerative conditions, but further work is required regarding this avenue of research.

**Figure 3:**
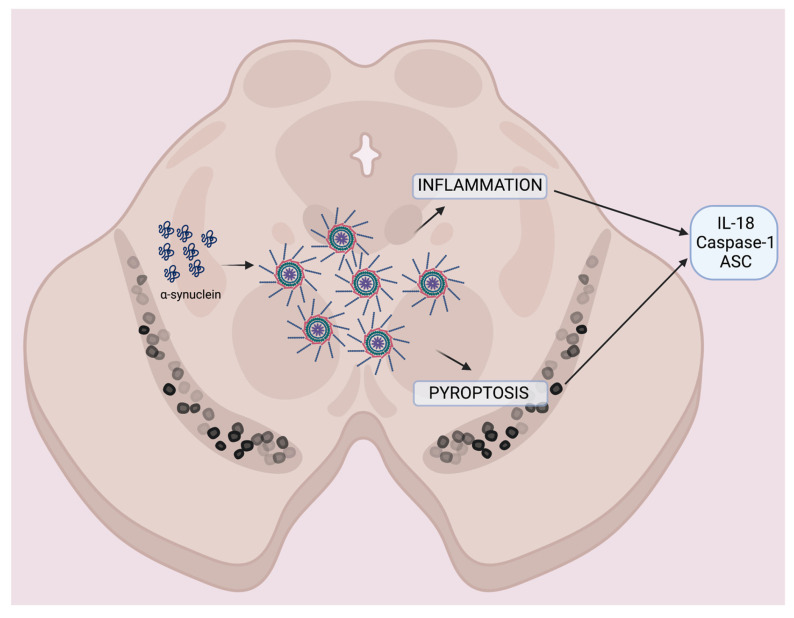
Model of the contribution of the inflammasome to PD pathology. (Figure created with BioRender).

Here, we proposed a model by which, in the SN, α-synuclein activates the inflammasome [23], resulting in inflammation mediated by the pro-inflammatory cytokine IL-18, which leads to inflammasome-mediated cell death (pyroptosis) [71]. Together, these factors lead to exacerbation of the inflammatory response present in PD, further accumulation of α-synuclein aggregates [16], and the release of caspase-1, ASC, and IL-18 that can be detected in the blood of patients with PD (Figure 3).

In conclusion, with the development of improved detection methods, the identification of resolute PD biomarkers has become feasible. Our study shows that the inflammasome signaling proteins caspase-1 and ASC and the pro-inflammatory cytokine IL-18 are reliable biomarkers for PD. We have established that inflammasome proteins and pro-inflammatory cytokines identified in the serum of patients with PD can serve as biomarkers. This opens the door for future validation and accessible diagnostic practices with the potential to monitor disease progression and outlook. Future studies need to examine whether the inflammasome proteins and associated pro-inflammatory cytokine biomarkers identified in this study may be used to distinguish between PD and other parkinsonian conditions, as well as to assess the ability of the candidate biomarkers to predict functional outcomes in clinical settings.

## 4. Materials and Methods

### 4.1. Participants

**Figure 4:**
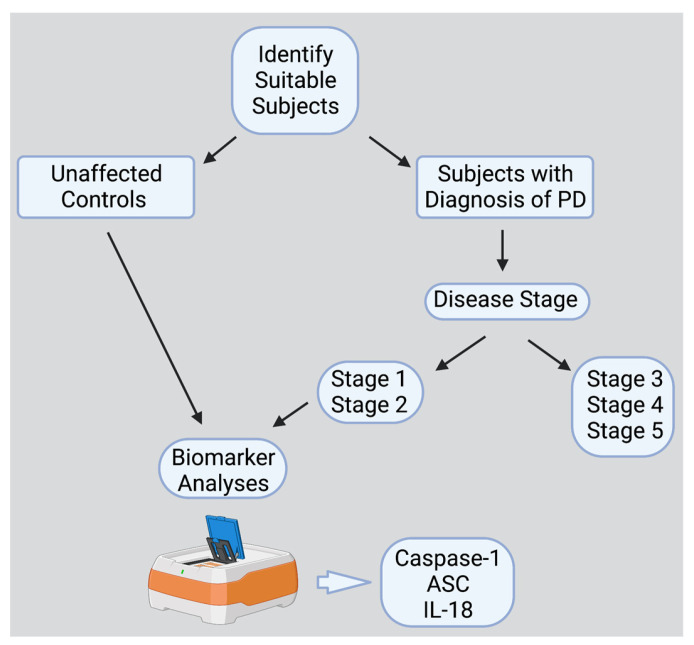
Flow Chart of the Study (Figure created with BioRender).

The PD subjects were enrolled in a study approved by The University of Miami’s Institutional Review Board (IRB protocol number 20070380), according to the ethical standards of the responsible Committee on Human Experimentation at the University of Miami and in compliance with the Declaration of Helsinki. Plasma was collected from EDTA blood tubes through centrifugation at 1800× *g* for 10 min at room temperature, within four hours of the blood being drawn and stored at −80 °C until analysis (Figure 4). PD plasma samples were obtained from 32 subjects (22 males (69%) and 10 females (31%)) with an age range of 41 to 82 years (median age of 66 years) (Table 4).

In the PD cohort, disease duration at the time of sample acquisition ranged from the year of diagnosis to 22 years after diagnosis (median duration of 7 years). The Hoehn and Yahr Scale was used for Parkinson’s disease progression evaluation, with 8 patients at stage 1 (25%) and 24 at stage 2 (75%). Of our patient cohort, 8 individuals (25%) received deep brain stimulation treatment and 24 did not (75%). Plasma samples collected in EDTA tubes from healthy age-matched unaffected controls were purchased from BioIVT. Samples from healthy controls were centrifuged at 1300× *g* for 10 min at room temperature within 1 h of collection. Healthy unaffected controls had an age range of 55 to 67 years (median age of 58 years), with a similar sex distribution between males (9, 56%) and females (7, 44%).

### 4.2. Simple Plex Assay

The Ella System (ProteinSimple, San Jose, CA, USA) was used to quantify inflammasome protein (caspase-1, ASC, and IL-18) concentrations from plasma samples from 32 patients with PD and 16 healthy age-matched controls, as described by the authors in [67]. Briefly, 30 μL of the sample was diluted in an equal amount of diluent reagent and loaded into 50 μL wells of a CART. Washing buffer was added to separate 1 mL wells in the same CART. Serum concentration was quantified via the Runner Software (version 3.8.012, San Jose, CA, USA) and further analyzed through the Simple Plex Explorer (version 3.8.0.12, San Jose, CA, USA).

### 4.3. Statistical and Biomarker Analysis

Simple Plex assay data were analyzed with PRISM 9 (GraphPad, Boston, MA, USA). The robust regression and outlier removal (ROUT) procedure (Q set to 1%) identified and removed outliers before further analysis was completed. Descriptive statistics were then obtained once normality was assured with the Shapiro–Wilk test or the D’Agostino and Pearson Test. Two-tailed *t*-tests and two-tailed Mann–Whitney U tests were selected to analyze parametric and non-parametric data, respectively. A *p*-value of <0.05 indicated statistical significance.

The AUC was calculated using ROC to further characterize biomarker potential and obtain information on specificity, sensitivity, cut-off points, and likelihood ratio. The cut-off point was defined as the highest likelihood of reliability vs. 1-specificity plot for each specific analyte. Higher sensitivity was prioritized over specificity to ensure a greater likelihood of reliability. Overall assay accuracy was measured, as well as the positive and negative predictive values.

RStudio/RMarkdown (version 1.2.5033) was used for linear regression analysis to explain the contribution of inflammasome protein levels to IL-18 expression. A stepwise regression procedure was completed using caspase-1 and ASC to predict IL-18 protein levels based on the lowest Akaike information criterion (AIC). Estimate, standard error, and *p*-values were then obtained for each of the predictors and intercepts of the linear regression. The Durbin Watson (DW) statistic was used to evaluate the model for autocorrelation. Residuals, mean of residuals, root mean square error (RMSE), confidence intervals, and the Bayesian information criterion (BIC) for the best fit model were also calculated.

## Figures and Tables

**Table 1:** ROC Analysis.

Biomarker	Area	Std. Error	95% C.I.	*p*-Value
Caspase-1	0.9567	0.03207	0.8939 to 1.0	<0.0001
ASC	0.8711	0.5714	0.7591 to 0.9831	<0.0001
IL-18	0.8535	0.0640	0.7281 to 0.9790	<0.0001

**Table 2:** Cut-off point in plasma of patients with PD.

Biomarker	Cut-Off Point (pg/mL)	Sensitivity (%)	Specificity (%)	LR	PPV (%)	NPV (%)	Accuracy (%)
Caspase-1	>1.450	96.88	84.62	6.297	94	92	93
ASC	>222.5	96.88	75	3.875	89	92	90
IL-18	>159.5	90.63	75	3.625	88	80	85

**Table 3:** Linear regression model to predict IL-18 level.

IL-18	Estimate	Std. Error	*p*-Value	Confidence Interval
Intercept	907.1865	321.7597	0.00858	249.114 to 1565.259
Caspase-1	60.6652	10.2042	1.85 × 10^−6^	39.795 to 81.535
ASC	−2.7946	0.9669	0.00722	−4.772 to −0.817
Adjusted R^2^		0.7768		
*p*-value		1.366 × 10^−10^		
BIC		527.844		
RMSE		781.5156		
Mean of residuals		4.88 × 10^−15^		
DW Statistic				
rho ! = 0			0.896	
rho < 0			0.444	
rho > 0			0.582	

**Table 4:** Characteristics of patients included in the study.

Sex	Males	22 (69%)
	Females	10 (31%)
Age (Range)		41 to 82
Age (Median)		66 years
Disease Duration (Median)		7 years
Hoehn and Yahr Scale	Stage 1	8 (25%)
	Stage 2	24 (75%)
Deep Brain Stimulation		8 (25%)

## Data Availability

Available data will be provided upon request to the corresponding author.

## Supplementary Materials

The following supporting information can be downloaded at: https://www.mdpi.com/article/10.3390/ph16060883/s1, Figure S1: Residual analysis results for the model IL-18~Caspase-1 + ASC.
