# Supplementary figures and images for: Proof-of-Principle Study of Inflammasome Signaling Proteins as Diagnostic Biomarkers of the Inflammatory Response in Parkinson’s Disease
Source: Pharmaceuticals (Basel). 2023 Jun 15;16(6):883. doi: 10.3390/ph16060883 (PMC10305113; doi:10.3390/ph16060883)

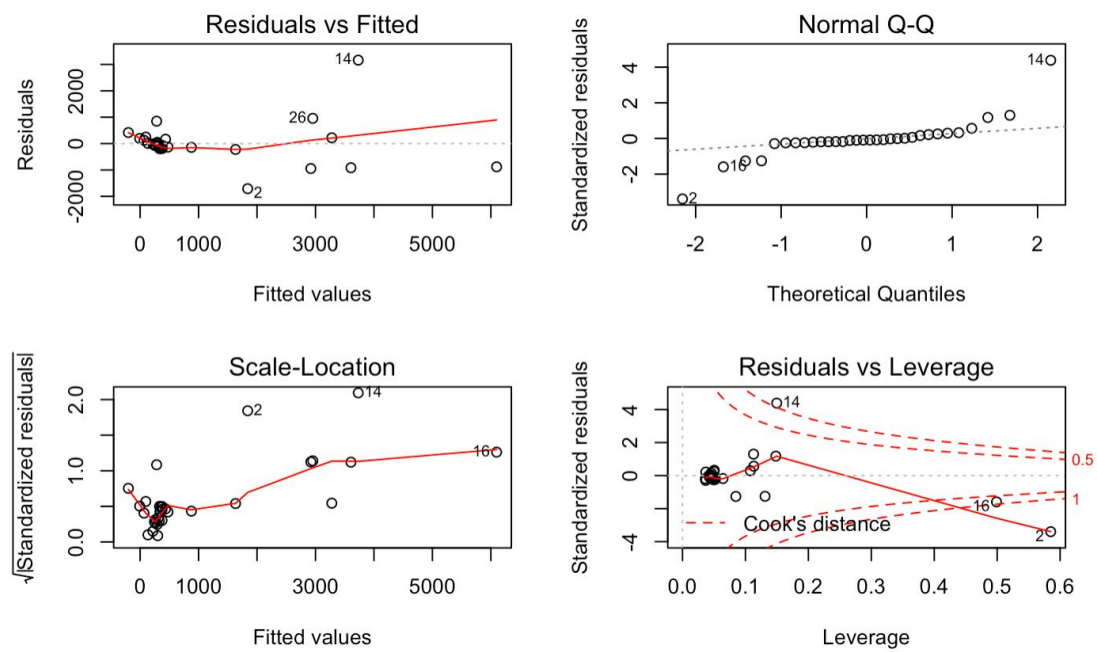

**Figure S1.** Residual analysis results for the model IL-18-Caspase-1 + ASC.

Supplement: Supplementary file 1 [file pharmaceuticals-16-00883-s001.zip › pharmaceuticals-2388876-supplementary.pdf]
